# Surface Chemistry of Ru/CeO_2_ Catalyst as Revealed by CO and ^15^N_2_ IR Probe Molecules

**DOI:** 10.1021/acs.langmuir.6c00814

**Published:** 2026-04-23

**Authors:** Oleksii Bezkrovnyi, Nikola Drenchev, Piotr Kraszkiewicz, Michael Vorochta, Iva Matolínová, Tcvetomir Venkov, Mirosława Pawłyta, Leszek Kepinski, Konstantin Hadjiivanov

**Affiliations:** † W. Trzebiatowski Institute of Low Temperature and Structure Research, Polish Academy of Sciences, Wroclaw 50-422, Poland; ‡ Institute of General and Inorganic Chemistry, Bulgarian Academy of Sciences, Sofia 1113, Bulgaria; § Centre of Mechatronics and Clean Technologies, Sitnyakovo Campus, Sofia 1113, Bulgaria; ∥ Department of Surface and Plasma Science, Faculty of Mathematics and Physics, 37740 Charles University, V Holešovičkách 2, Prague 8, Prague 180 00, Czechia; ⊥ Materials Research Laboratory, 49569 Silesian University of Technology, Gliwice 44-100, Poland; # Institute of Nuclear Physics, Polish Academy of Sciences, ul. Radzikowskiego 152, Kraków 31-342, Poland

## Abstract

A comprehensive molecular-level characterization of the surface chemistry of Ru/CeO_2_ was performed using FTIR spectroscopy of two complementary probe molecules: CO, the most commonly used IR probe for cationic and metal sites, and ^15^N_2_, an inert molecule that selectively detects only the strongest adsorption centers. Blank experiments with bare ceria octahedra were also performed. The effects of oxidative and reductive pretreatments on CO and ^15^N_2_ adsorption were systematically examined for both Ru/CeO_2_ and CeO_2_. The results show that the oxidized catalyst contains an oxidized Ru–oxide/hydroxide phase covering the metal particles and part of the support surface, thus completely blocking metallic ruthenium sites and markedly reducing the number of available CO adsorption sites on ceria and completely suppressing ^15^N_2_ adsorption. This blocking effect is much more pronounced on the highly reactive {110} and {100} facets than on the more stable {111} surface. After reduction, metallic Ru becomes exposed, and some previously inaccessible ceria sites are restored. Structural and electronic characterization using XRD, STEM, H_2_-TPR, and NAP-XPS provides key complementary information on the morphology, composition, reducibility, and chemical state of the catalysts, enabling consistent interpretation of the IR spectroscopic results.

## Graphical abstract

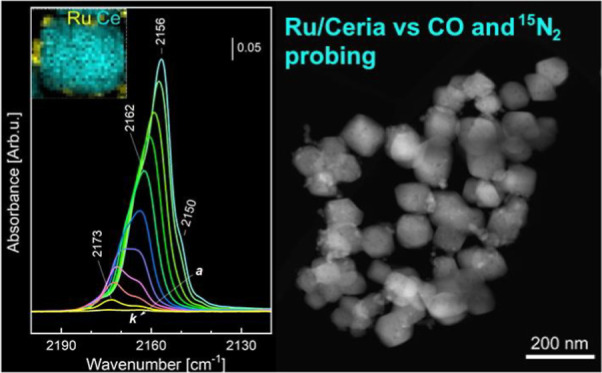

## Introduction

The catalytic efficiency of ruthenium supported on cerium oxide (Ru/CeO_2_) has attracted increasing attention in recent years for two key reasons. First, it is an effective multifunctional catalyst for an extensive range of redox reactions, like propane oxidation,^1^ methane steam reforming,^2^ formaldehyde oxidation,^3^ ethanol dehydration,^4^ CO_2_ methanation^5^ etc. Second, Ru/CeO_2_ has a lower price compared with frequently used precious (Pt and Pd) metal-based catalysts. The key factor responsible for the catalytic activity and stability of ceria-based catalysts is the reversible Ce^4+^/Ce^3+^ transition in ceria.^6^ This process enhances the catalysts’ ability to transport oxygen to the active sites, an essential step in the Mars-van Krevelen (MvK) reaction mechanism.^6^ In M/CeO_2_ (M = metal) systems, the actual “active site” is rarely just the metal atom/cation alone, but rather a synergistic ensemble formed at the metal-support interface, often modulated by the support’s facet, defect concentration, and metal loading. Due to high sensitivity to the gas-phase environment (oxidizing or reducing), the morphology (single atoms and/or nanoparticles)^7,8^ and oxidation state of the Ru species^9,10^ are dynamic. Using a combination of in situ Raman and DRIFTS techniques, Xu et al.^11^ observed that the reversible transformation of Ru species supported on CeO_2_ depends on the reaction atmosphere and that these structural changes strongly influence CO oxidation activity. Khivantsev et al.^12^ showed that atomically dispersed Ru^2+^ ions on CeO_2_ are highly active in NO oxidation and NO_*x*_ storage.

Recently, using the NAP-XPS technique, we showed that Ru NPs oxidized to the volatile RuO_4_ form upon Ru/CeO_2_ exposure to the oxygen-rich atmosphere at ≥300 °C.^9,10,13^ On the one hand, this leads to Ru loss from the catalyst; on the other hand, it creates favorable conditions for the redispersion of Ru nanoparticles via the gas phase. Thus, it is logical to assume that two forms of Ru could be present during the oxidative pretreatment of Ru/CeO_2_: RuO_*x*_ nanoparticles and atomically dispersed Ru on the ceria support. Despite the relatively low mass contribution of the hypothetical atomic Ru sites, they could significantly affect the gas adsorption processes on Ru/CeO_2_ catalysts. To verify this hypothesis, we tested the surface of oxidatively and reductively treated CeO_2_ and Ru/CeO_2_ catalysts using two IR probe molecules: CO and ^15^N_2_. Carbon monoxide (CO) has been the most widely used probe molecule, providing information on the oxidation and coordination states of the metal/cationic sites.^14,15^ However, CO can reduce Ce^4+^ to Ce^3+^ ^16–18^ and change the oxidation state and dispersion of ruthenium,^14,19,20^ thereby distorting the data interpretation. For this reason, ^15^N_2_ was chosen as a second “inert” probe molecule, which is unlikely to induce significant surface perturbations and thus can control the CO adsorption results. The application of the ^15^N_2_ isotopologue enabled the avoidance of interference from the overlapping atmospheric CO_2_-related signal and N_2_ itself. Previously, this approach has been successfully used to probe the surface of CeO_2_ nanoparticles.^21,22^ Thus, our work aimed to investigate how the type of pretreatment and the presence of Ru nanoparticles influence both the number and the nature of the adsorption sites for the CO and ^15^N_2_ probe molecules on CeO_2_ support. Also, the usability of CO and ^15^N_2_ probe molecules to test the surface of Ru NPs was investigated. NAP-XPS was used as the reference technique to explore the chemical state of the Ru/CeO_2_ catalyst—necessary for the correct interpretation of IR gas adsorption data.

### Experiment

The support (CeO_2_ nanooctahedra) was synthesized by microwave-assisted hydrothermal method (MWHT).^23,24^ 0.433 g Ce­(NO_3_)_3*_6H_2_O was first dissolved in 35 mL distilled water. The obtained solution was mixed with 5 mL of an aqueous solution of 5 mg of sodium phosphate (Na_3_PO_4_), stirred for 30 min, and heated at 200 °C for 1 h under autogenous pressure in an MWHT autoclave. The as-obtained CeO_2_ precipitate was washed, dried at 60 °C for 12 h, and calcinated in air at 900 °C for 3 h. Next, 500 mg of the support was ultrasonically dispersed in 50 mL of H_2_O and an appropriate amount of ruthenium­(III) nitrosylnitrate solution containing 11 wt % Ru was added to the ceria suspension to obtain a 2.5 wt % Ru/CeO_2_ catalyst, which was then ultrasonically treated for 30 min. After that, a few drops of ammonia solution (30% in water) were added to increase the pH from 1.5 to 9.5, and the suspension was stirred for 30 min. The obtained suspension of Ru/CeO_2_ was centrifuged. Precipitates were washed five times with deionized water, dried at 60 °C for 12 h, and annealed in H_2_ at 500 °C for 3 h.

The crystal structure of the samples was determined by powder X-ray diffraction (XRD) using an X’Pert PRO PANalytical diffractometer with Cu Kα radiation. Calculations of lattice parameters, average crystallite size, and average strain were performed using Rietveld refinement with the FullProf program (version November 2023).^25^ The Thompson-Cox-Hastings (TCH) pseudo-Voigt function was applied to fit the diffraction profiles, enabling the estimation of size and strain broadening contributions.

The morphology of the samples and element distribution mapping were studied by transmission electron microscopy (probe-corrected FEI TITAN microscope operating at 300 kV). The general chemical composition of the samples was verified by energy dispersive X-ray spectroscopy (EDS) using a FEI Nova NanoSEM 230 instrument equipped with an EDAX Genesis XM4 detector. The reducibility of the samples was studied using temperature-programmed reduction (H2-TPR), which involved heating the samples at a rate of 10 °C/min to 1000 °C in a H_2_/Ar (5 vol %) flow (30 mL/min). The H_2_ consumption was monitored by a thermal conductivity detector (TCD). Before H_2_-TPR analysis, CeO_2_ and Ru/CeO_2_ samples were pretreated in air flow at 300 °C to oxidize them to the stoichiometric state and reduce the number of surface –OH groups—a source of the distortion of the TCD signal.

NAP-XPS measurements were performed using a NAP-XPS system (SPECS GmbH) equipped with a monochromatized Al Kα X-ray source (μFOCUS 600), a differentially pumped hemispherical electron energy analyzer (PHOIBOS 150 NAP), and a high-pressure cell (DeviSIM NAP). Samples were initially analyzed under ultrahigh vacuum (UHV) at room temperature, recording survey spectra as well as the Ce 3d, Ru 3d, and O 1s regions. Subsequently, the same regions were measured in the presence of H_2_ and O_2_ at 1 mbar while the samples were stepwise annealed from 25 to 300 °C. Sample heating was achieved by contact with a hot sample stage, which was heated via an e-beam with an energy of 1 kV (installed at the backside of the NAP cell). The Ce^3+^ percentage was calculated using a procedure proposed by Bezkrovnyi et al.^26^ It has been used in our previous NAP-XPS studies on ceria-based catalysts.^15,27,28^ The Ru 3d fitting procedure was performed as reported in our previous study.^10^

The FTIR spectra were recorded with a Thermo Scientific Nicolet 6700 FTIR spectrometer equipped with an MCT-A detector. The accumulated scans were 64 and the spectral resolution was 2 cm^–1^. Self-supporting pellets (ca. 20 mg cm^–2^) were prepared by pressing sample powder and treated directly in a purpose-made IR cell. It allowed measurements between ambient temperature and −173 °C and was connected to a vacuum-adsorption apparatus with a residual pressure below 10^–3^ Pa. The treatment of the obtained spectra (background and gas-phase corrections) was performed using OMNIC spectroscopy software (version 9).

Prior to the IR adsorption experiments, the CeO_2_ and Ru/CeO_2_ samples were activated in O_2_ (Messer, >99.999%) at the desired temperature (typically 300 °C), followed by a 1 h vacuum treatment at 300 °C. The reduction was conducted at 400 °C for the CeO_2_ sample and at 300 °C for the Ru/CeO_2_ sample in the presence of 55 mbar H_2_ (Messer, >99.999%) for 1 h. CO (Merck, 99.5%) and ^15^N_2_ (Aldrich, 98 at. %) were adsorbed at −173 °C (100 K).

## Results and Discussion

### Structure and Morphology

As seen in Figure 1a,b, the as-obtained Ru/CeO_2_ sample contains octahedral-shaped ceria particles decorated with Ru NPs, a few nanometers in size. In our previous study, we showed that ceria nanoparticles obtained by the described method are single crystals, mainly terminated by {111} facets.^13,23,24^ The EDS analysis shows that overall Ru content in the as-obtained Ru/CeO_2_ sample was 2.7 wt %. Element distribution mapping presented in Figure 1b (inset) shows that most of the ruthenium is present in the form of Ru nanoparticles; the Ru-related signal from the ceria support is negligible. It indicates the minor scale of Ru dissolution into the ceria support.

**Figure 1:**
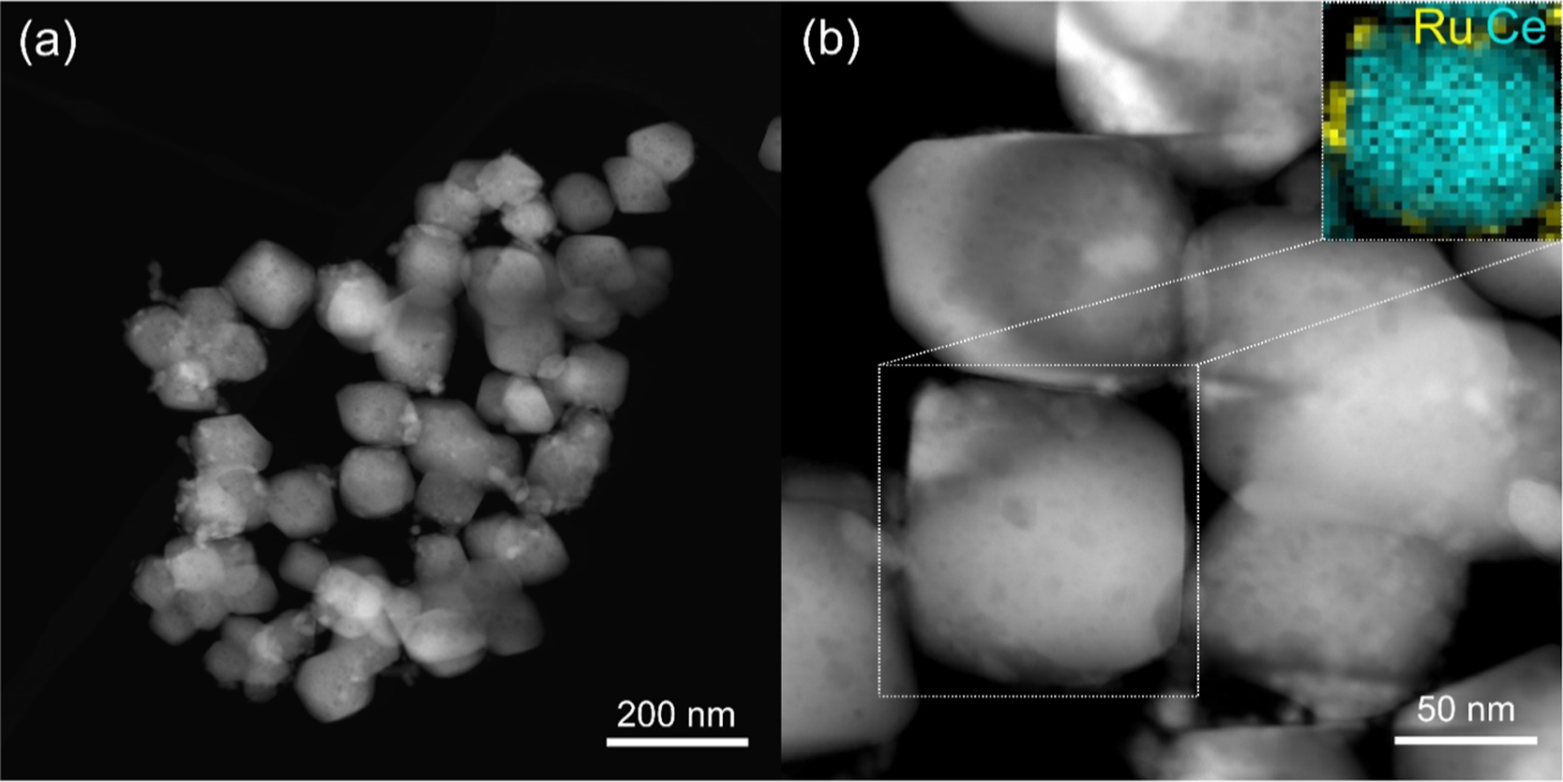
Representative low (a) and middle (b) magnification STEM images of the as-obtained Ru/CeO_2_ sample. In the inset—EDS map of Ce and Ru distribution.

The powder XRD patterns of as-prepared CeO_2_ showed only reflections corresponding to the fluorite-type CeO_2_ structure with space group *Fm*-3*m*. In the case of Ru/CeO_2_, an additional, very weak reflection related to the Ru hexagonal phase (space group *P*6_3_/*mmc*) was observed (Figure 2). The average ceria crystallite sizes of the as-prepared CeO_2_ and Ru/CeO_2_ samples were calculated to be 37 and 41 nm, respectively (see Table S1 in the Supporting Information). The average size of Ru nanocrystals in the Ru/CeO_2_ sample was 14 nm.

**Figure 2:**
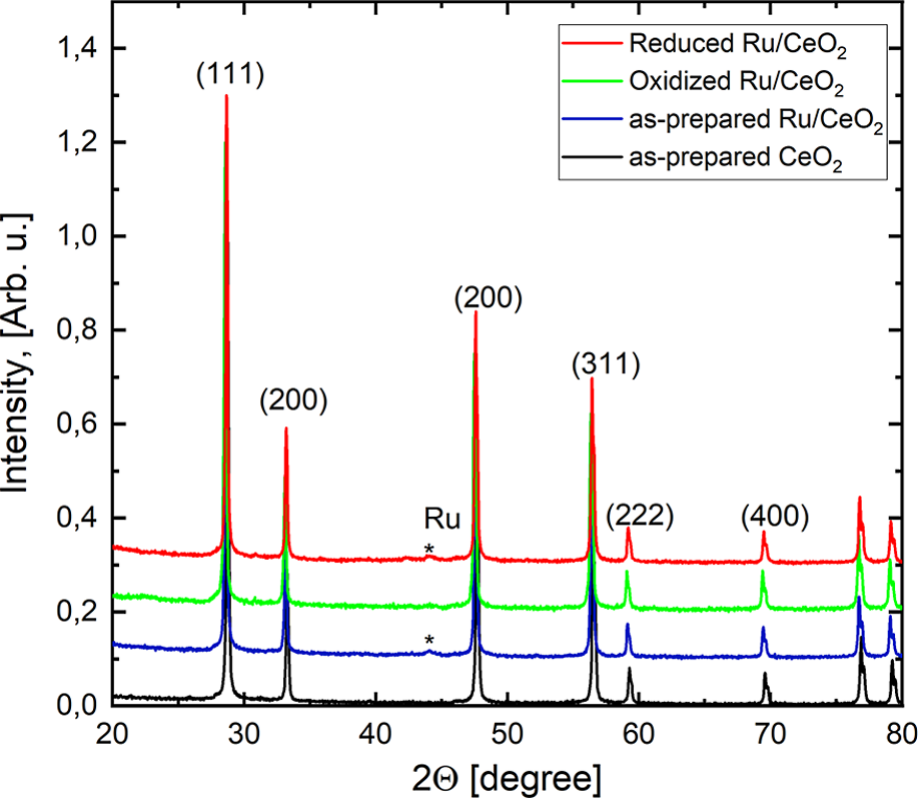
Powder XRD patterns of the CeO_2_ and Ru/CeO_2_ samples.

Considering the reductive environment used during the synthesis of Ru/CeO_2_, an additional treatment in air at 300 °C was performed to restore the stoichiometric state (this sample is called below as “oxidized-Ru/CeO_2_”). Next, the oxidized Ru/CeO_2_ sample was reduced by treating in H_2_/Ar (5 vol %) flow (30 mL/min) at 300 °C for 1 h; this sample is hereafter referred to as “reduced-Ru/CeO_2_”. Powder XRD analysis shows that the “oxidized Ru/CeO_2_” sample exhibits the reflections corresponding to the CeO_2_ support only (c.f. Table S1). No peaks related to metallic ruthenium or ruthenium oxides were detected. Most likely, this is due to the partial oxidation of Ru nanoparticles, resulting in the formation of a RuOx shell. Indirectly, this explanation is supported by NAP-XPS data presented below. XRD pattern of the reduced-Ru/CeO_2_ shows strong reflections corresponding to the fluorite-type CeO_2_ structure. A weak peak related to metallic Ru hexagonal phase was also observed (see Table S1), indicating at least partial reduction of oxidized RuO_*x*_ forms.

### H_2_-TPR and NAP-XPS Study

The thermal reducibility of the as-prepared CeO_2_ and Ru/CeO_2_ samples upon their exposure to H_2_ was studied by H_2_-TPR. As shown in Figure 3, the CeO_2_ support exhibits a dominant TPR peak at 825 °C and several weaker signals with a main feature at 407 °C. In agreement with numerous literature reports,^29–36^ the TPR peaks around 407 °C are assigned to reduction of surface Ce^4+^ cations while the peak at 825 °C is attributed to bulk reduction. The TPR profile of Ru/CeO_2_ above 600 °C closely matches that of bare ceria. This is consistent with the literature, which shows that supported metals do not influence the bulk reduction of ceria.^32–36^ In contrast, the profile in the low-temperature region changes dramatically upon Ru deposition. The surface-reduction peaks characteristic of ceria disappear, and two new features emerge: a sharp, intense peak at 25 °C and a weaker one at 260 °C. Such behavior mirrors previous observations for various supported metals, such as Rh,^32,33,36^ Pt,^34,36^ and Ag,^35^ implying they promote ceria surface reduction and shift the corresponding peaks to substantially lower temperatures.

**Figure 3:**
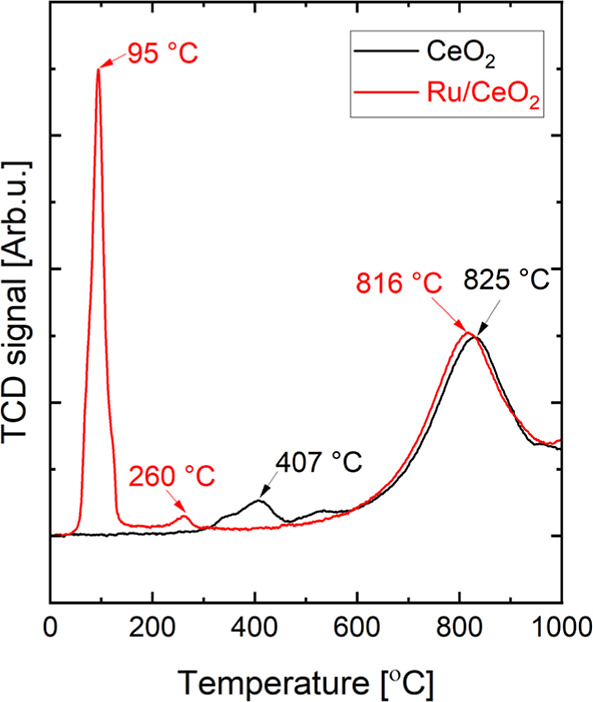
H_2_-TPR patterns of CeO_2_ and Ru/CeO_2_ samples.

The strong peak at 95 °C is attributed to the reduction of Ru oxide species together with the facilitated reduction of nearby surface Ce^4+^ sites. This is most plausibly explained by H_2_ dissociation on Ru nanoparticles followed by hydrogen spillover onto ceria.^2,10^ The weaker peak at 260 °C likely corresponds to the reduction of some Ru species or of surface Ce^4+^ sites located farther from Ru particles and therefore less affected by the spillover process. Experimental and calculated values of H_2_ consumption are collected in Table S2 in the Supporting Information.

The evolution of the chemical state of the Ru/CeO_2_ catalysts during the redox cycle was investigated using NAP-XPS. Figure 4a,b show the Ce 3d and Ru 3d regions of the NAP-XPS spectra, respectively.

**Figure 4:**
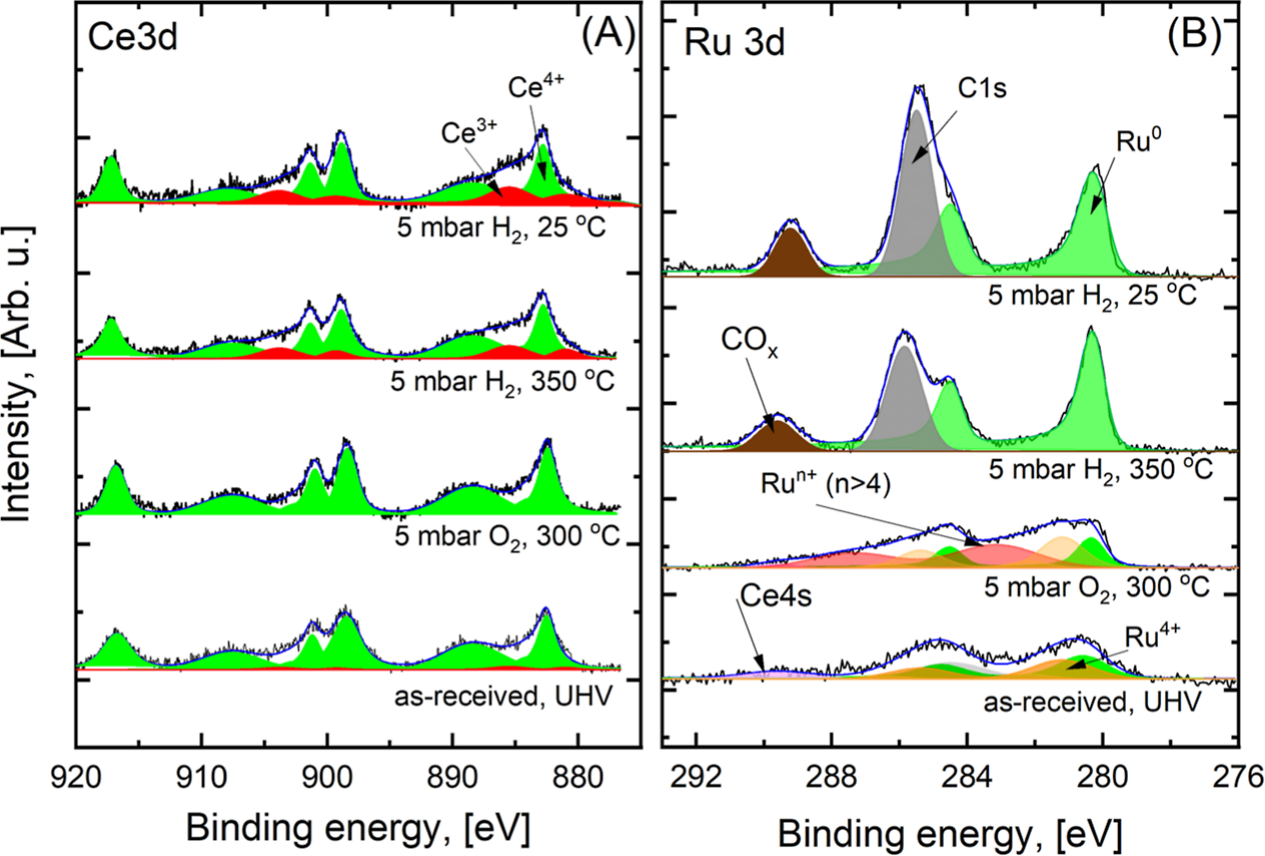
NAP-XPS Ce 3d (A) and Ru 3d (B) spectra of the Ru/CeO_2_ sample recorded under different treatments. The measured spectra are shown in black. The Ce 3d region was deconvoluted into Ce^3+^ (red) and Ce^4+^ (green) components, while the Ru 3d region was deconvoluted into Ru^0^ (green), Ru^4+^ (orange), Ru^*n*+^ (*n* > 4), as well as contributions from C 1s (gray) and Ce 4s (violet).

As seen in Figure 4, the CeO_2_ support in the as-prepared Ru/CeO_2_ sample is almost fully oxidized. Ru nanoparticles also exhibit a high degree of oxidation, with 38% of metallic Ru and the remaining 62% corresponding to RuO_2_. Exposing the Ru/CeO_2_ sample to 5 mbar O_2_ and heating to 300 °C results in its almost complete oxidation. No Ce^3+^ related doublets were detected, and most of Ru existed as Ru^4+^ and Ru^n+^ (*n* > 4+) with a minor amount of Ru^0^ states (Figure S1 from Supporting Information). Increasing the temperature to 350 °C in 5 mbar H_2_ leads to a noticeable reduction of ceria support (approximately 20% of Ce^3+^) and complete reduction of Ru nanoparticles to the metallic state (Figure 4). The carbon detected likely originates from the residual contamination of the NAP-cell that transferred to the sample upon reductive atmosphere at elevated temperatures. Cooling the Ru/CeO_2_ sample to room temperature under 5 mbar H_2_ does not alter its chemical state relative to its state at 350 °C in H_2_.

### In-Situ IR Studies

#### Background IR Spectra

The IR spectra of the ceria support and the Ru/CeO_2_ catalyst recorded after oxidative and reductive treatments are shown in Figure 5A. To activate the samples, we used the oxidative preheating at 300 °C in air for 1 h, followed by evacuation at the same temperature.

**Figure 5:**
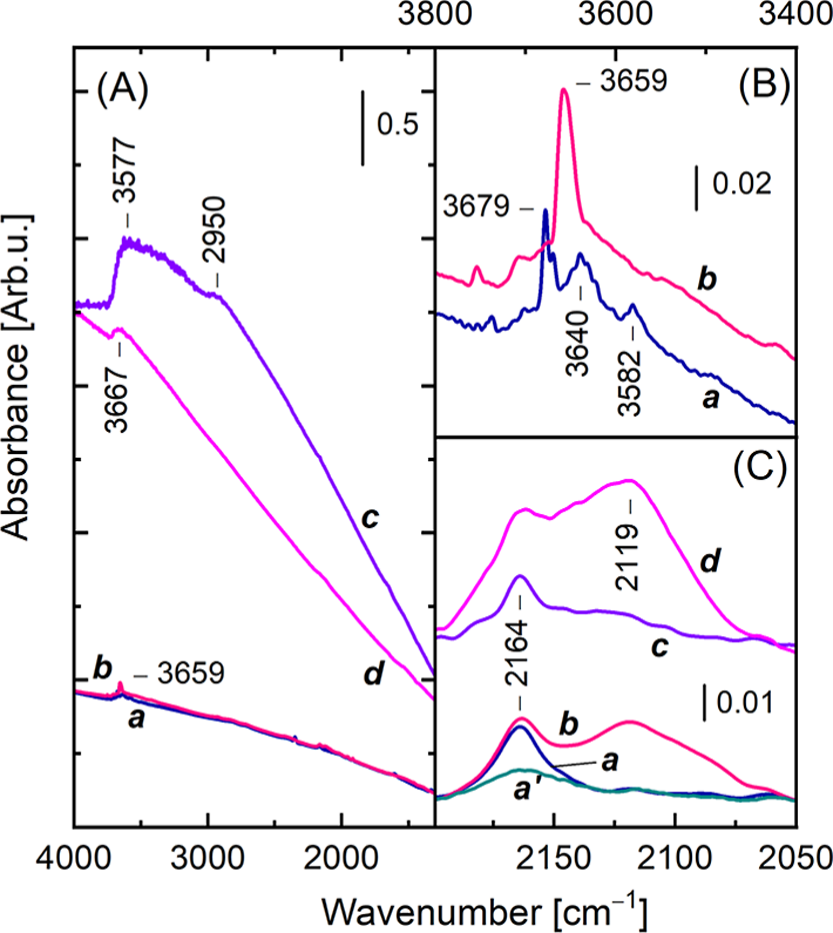
FTIR spectra of the samples studied: CeO_2_ activated at 300 °C (a) and at 400 °C (a’) and after H_2_ reduction at 300 °C (b); Ru/CeO_2_ activated at 300 °C (c) and reduced with H_2_ at 300 °C (d). Panel (A) shows a general overview, panel (B) is an expanded view of the spectra of ceria in the ν­(OH) region and panel (C) displays the region of the Ce^3+^ electronic transition—these spectra are slope-corrected.

Three bands are observed in the spectrum of activated ceria in the hydroxyl region: at 3679, 3640, and 3582 cm^–1^ (Figure 5B). Although hydroxyl vibrations in ceria depend strongly on morphology, bands at similar positions have been reported previously.^21,27–42^ The bands at 3679 and 3640 cm^–1^ are attributed to bridging OH groups, while the weak band at 3582 cm^–1^ probably originates from triply bridging hydroxyls.

A careful inspection of the 2200–2000 cm^–1^ region (Figure 5C) reveals a weak band at 2164 cm^–1^. The ^2^F_5/2_ → ^2^F_7/2_ spin–orbit electronic transition of Ce^3+^ is typically observed between 2130 and 2100 cm^–1^ for ceria.^21,31,38,41–48^ However, this transition can be observed at higher frequencies.^44,45,49^ In a recent study,^49^ we showed that UiO-66-Ce exhibits a band at 2153 cm^–1^ assigned to Ce^3+^ sites displaying high resistance toward reoxidation, an effect attributed to stabilization by organic ligands. The similar behavior and comparable frequency of the band observed in the present study support its tentative assignment to a Ce^3+^ electronic transition associated with Ce^3+^ sites stabilized by residual carbonaceous species. This interpretation is further supported by the strong attenuation of the band after oxidation at 400 °C (spectrum a’), indicating oxidation of most of the Ce^3+^ sites.

The spectrum of the reduced ceria sample is broadly similar to that of the activated material (Figure 5A, spectrum b). However, a new band appears at 2119 cm^–1^, characteristic of the Ce^3+^ electronic transition in reduced CeO_2_, confirming that reduction proceeds under the applied conditions (Figure 5C). It agrees well with both H_2_-TPR data for pristine ceria nanoparticles presented below (see Figure 3), and our previous H_2_-TPR and NAP-XPS results, which confirms the surface Ce^4+^ → Ce^3+^ reduction at ≥300 °C.^29,46,47^ Changes are also observed in the OH region (Figure 5B): the main band shifts to 3659 cm^–1^, a position typical of partly reduced ceria.^40,41^

The spectrum of activated Ru/CeO_2_ (Figure 5, spectrum c) closely resembles that of the CeO_2_ support in the 2200–2000 cm^–1^ regions. A weak Ce^3+^ band at 2164 cm^–1^ is again detected with almost identical intensity. At higher frequencies, the spectrum shows a broad halo extending below 3600 cm^–1^, indicating the presence of H-bonded hydroxyls.^48^ Although their precise nature cannot be established, they are clearly associated with the presence of Ru. Notably, no bands attributable to Ru=O species (expected overtone near 1900 cm^–1^) are detected.^50,51^ This seems to contradict the NAP-XPS and H_2_-TPR data, which show the noticeable RuO_2_ fraction in the oxidized Ru/CeO_2_ sample. Moreover, the temperature and duration of the oxidative treatment in NAP-XPS and H_2_-TPR, as well as during the activation stage before the IR experiment, were similar: 300 °C for approximately 1 h. This apparent contradiction between the NAP-XPS, H_2_-TPR, and IR data can be explained by the fact that, the RuO_2_ phase is highly hydroxylated, which results in the lack of distinct Ru=O bonds, a state stable even after evacuation at 300 °C. This hypothesis is supported by the CO adsorption results (see below).

Separate experiments were performed to check the stability of the ruthenium oxo-hydroxy phase. A sample was evacuated at increasing temperatures and the resulting spectra are shown in Figure S2 of the Supporting Information. It is seen that, although decreasing in intensity, the band due to H-bonded hydroxyls is still present after 500 °C evacuation, showing the high stability of the oxy-hydroxide phase.

The broad hydroxyl feature vanishes upon reduction, leaving only a weak band near 3600 cm^–1^, suggesting that these OH groups were located on or near the Ru nanoparticles and mostly were removed upon reduction of the surface shell of Ru^n+^ species.

Another effect of reduction is that the band at 2119 cm^–1^ sharply increases in intensity, indicating the formation of a significant fraction of reduced Ce^3+^ sites (Figure 5C, spectrum d). In agreement with the TPR results, this effect is more pronounced for Ru/CeO_2_ than for the pure support, confirming that supported Ru promotes ceria reduction.

All samples exhibit a broad band centered at approximately 1000 cm^–1^ (not shown), which remains essentially unchanged irrespective of the pretreatment applied. To our knowledge, this feature has not been explicitly discussed in the literature. Additional experiments showed that ceria with cubic morphology exhibits mainly a band at 738 cm^–1^ in the low-frequency region. After calcination at 650 °C, which induces partial transformation of {100} facets into thermodynamically more stable {111} facets, the intensity of the 738 cm^–1^ band decreases, while a set of bands around 1000 cm^–1^ appears (Figure S3 in the Supporting Information). These observations suggest that the bands near 1000 cm^–1^ are associated with vibrational modes characteristic of the {111} ceria surface, which dominates the morphology of our support. At this stage, however, we refrain from proposing a detailed assignment of the individual components.

In summary, activated ceria contains predominantly Ce^4+^, although a small amount of Ce^3+^ probably exists after activation at 300 °C, likely stabilized by carbonaceous residues. Reduction at 300 °C significantly increases the concentration of Ce^3+^ sites. In activated Ru/CeO_2_, cerium again occurs mainly as Ce^4+^, with a residual amount of Ce^3+^ similar to that of the bare support. Reduction leads to (i) a higher concentration of Ce^3+^ than in the pure support, and (ii) almost full disappearance of the H-bonded hydroxyls, likely due to the reduction of surface Ru^n+^ species.

### Choice of Probe Molecules

To test the adsorption sites on the surface of the materials studied, we used two probe molecules, CO and N_2_. CO is the most frequently used molecular probe, and there are many IR investigations on its adsorption on bare ceria^16–18,37,38,51–54^ and on supported ruthenium.^14,19,20^ The experiments were performed at—173 °C because of several reasons: (i) the interaction of CO with Ce^*n*+^ sites is weak and low temperature is required to detect all sites;^14^ (ii) at ambient temperature^16,17^ or even at −100 °C^18^ CO may reduce ceria, forming Ce^3+^ and carbonate-like species, which modify the surface; (iii) CO-induced oxidation of metal ruthenium particles may occur at ambient temperature and to produce nonclassical ruthenium carbonyls, Ru^*n*+^(CO)_*x*_.^14,19,20^

In contrast, N_2_ does not probe all Ce^*n*+^ sites even at low temperature because it interacts more weakly with metal cations than CO.^21,22^ The results of the present study indicate that this limitation also extends to ruthenium sites. Thus, N_2_ selectively probes only the strongest sites which complement the data of the CO adsorption experiments. Importantly, N_2_ does not modify the surface and therefore does not perturb the intrinsic properties of the samples. To avoid interference from atmospheric CO_2_, the ^15^N_2_ isotopologue was used for the experiments.

### Adsorption of CO on Oxidized and Reduced Ceria Support

The IR spectra of CO adsorbed on activated ceria are shown in Figure 6A. At low coverage, a band at 2173 cm^–1^ dominates in the spectrum, and it is attributed to CO adsorbed on the most electrophilic Ce^4+^ sites from the {110} crystal plane and some crystal edges.^37^ At high coverage, a band at 2168–2162 cm^–1^ develops and is attributed to CO adsorbed on the {100} plane. With further coverage increase, an intense band at 2156 cm^–1^ appears, assigned to CO adsorbed on the {111} plane, where the Ce^4+^ sites are less electrophilic. The intensity ratio between the bands is consistent with the fact that the most abundant plane in our sample is {111} (see Figure 1).

**Figure 6:**
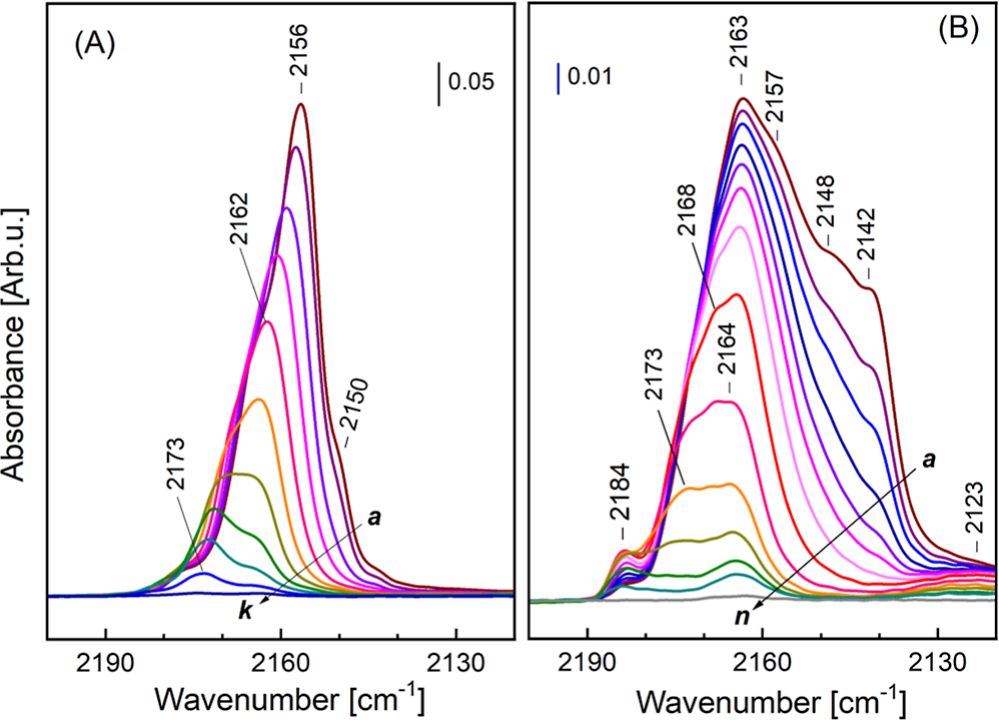
FTIR spectra of CO adsorbed at −173 °C on the CeO_2_ support. (A)—Oxidized sample. (B)—Reduced sample. Coverage decreases in the sequence (a–n).

At very high coverages, CO interacts with surface hydroxyls, giving rise to a band component around 2150 cm^–1^.^37,53^ Simultaneously, the OH bands are red-shifted with simultaneous widening and an increase in intensity (Figure S5), indicating formation of OH–CO adducts. No evidence for accessible Ce^3+^ was found, but the strong bands in the region could have masked eventual carbonyls of Ce^3+^ in low concentration.

Overall, these spectra indicate that surface cerium is predominantly in the Ce^4+^ oxidation state and that the {111} facets are the most exposed.

The next step was to study the reduced ceria sample. According to the TPR experiments (see Figure 2), only part of the surface Ce^4+^ sites should be reduced under the applied conditions. The spectra of CO adsorbed on the reduced sample (Figure 6B) differ significantly from those of the oxidized surface, confirming that partial reduction of Ce^4+^ sites has occurred. A weak band at 2184 cm^–1^, absent in the oxidized sample, appears at low coverage. This band cannot be attributed to Ce^3+^–CO species because they absorb at lower frequencies.^38^ Instead, it is assigned to CO bound to Ce^4+^ sites that have become more electrophilic upon local coordination loss due to oxygen removal—i.e., Ce^4+^ adjacent to an oxygen vacancy. The band decreases at higher CO coverages, indicating the formation of geminal Ce^4+^(CO)_2_ complexes most likely absorbing near 2173 cm^–1^. This assignment is consistent with the low coordination of the Ce^4+^ adsorption sites.

A band at 2142 cm^–1^ also emerges; a similar feature has been reported for CO bound to Ce^3+^ sites on the {111} surface.^38^ At very high coverages, a band at 2147 cm^–1^ appears, attributed primarily to OH··CO interactions, although some contribution from cerium carbonyls cannot be excluded. The magnitude of the OH band shift is smaller than for the oxidized sample, reflecting the lower acidity of OH groups associated with Ce^3+^ as compared to Ce^4+^.

The most intense band appears at 2163 cm^–1^ with a shoulder at 2157 cm^–1^. Carbonyls of Ce^3+^ on the {111} ceria facets have been reported to absorb at slightly higher frequency than those of Ce^4+^.^40^ Thus, the results indicate that some Ce sites on the {111} surface have been reduced.

In summary, CO adsorption on the reduced ceria demonstrates partial surface reduction and the coexistence of Ce^3+^ and Ce^4+^ sites, including the emergence of Ce^4+^ cations adjacent to oxygen vacancies having enhanced electrophilicity.

### Adsorption of ^15^N_2_ on Oxidized and Reduced Ceria

The spectra of ^15^N_2_ adsorbed on oxidized ceria (Figure 7A) show a main band around 2253 cm^–1^, attributed to adsorption on {100} facets, with a minor contribution from OH···N_2_ interactions at high coverage. This is corroborated by the red shift of the OH band at 3680 cm^–1^ (Figure S4). A shoulder near 2260 cm^–1^ is attributed to ^15^N_2_ species bound to sites on {110} facets and at crystal edges, and the intensity ratio of these bands is consistent with the CO adsorption data. As known from previous studies,^22^ Ce^4+^ cations on {111} are too weakly acidic to form adducts with N_2_.

**Figure 7:**
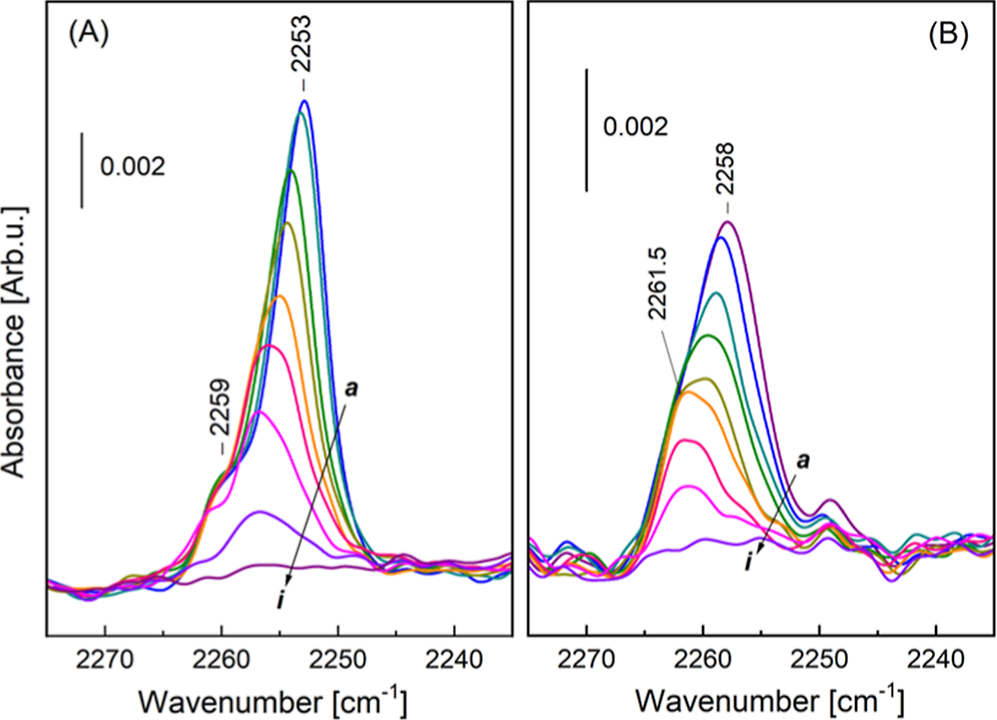
FTIR spectra of ^15^N_2_ adsorbed at −173 °C on the CeO_2_ support. (A)—Oxidized sample. (B)—Reduced sample. Coverage decreases in the sequence (a–i).

When ^15^N_2_ was adsorbed on the reduced sample (Figure 7B), the total intensity decreased by a factor of ∼4. The dominant band at 2258 cm^–1^ is associated with nonreduced surface Ce^4+^ sites. A weaker band at 2262 cm^–1^ indicates the presence of Ce^4+^ sites with enhanced electrophilicity, i.e., Ce^4+^ adjacent to oxygen vacancies, consistent with the CO results.

No bands attributable to ^15^N_2_ bound to Ce^3+^ sites (expected at ∼2155 cm^–1^)^21^ were distinguished, likely because they are masked by the nearby 2158 cm^–1^ feature. No interactions with OH groups were observed, consistent with the low acidity of surface OH species on reduced ceria.

### Adsorption of CO on Oxidized and Reduced Ru/CeO_2_

CO adsorption on the oxidized Ru/CeO_2_ produces a relatively simple spectrum (Figure 8A), dominated by a band at 2159 cm^–1^ with a low-frequency shoulder. Notably, no bands appear near 2173 or 2164 cm^–1^, indicating that the Ce^4+^ sites on the {110} and {100} facets are entirely blocked by deposited ruthenium. Also, it cannot be ruled out that the CO adsorption sites on the low exposed (100) and (110) planes have been initially blocked because we detect them neither on oxidized, nor on reduced sample. The existence of H-bonded hydroxyls (although few) on the reduced sample could suggest that Ru on (110) and (100) remains in an oxidized state and thus behaves identically after the two pretreatments.

**Figure 8:**
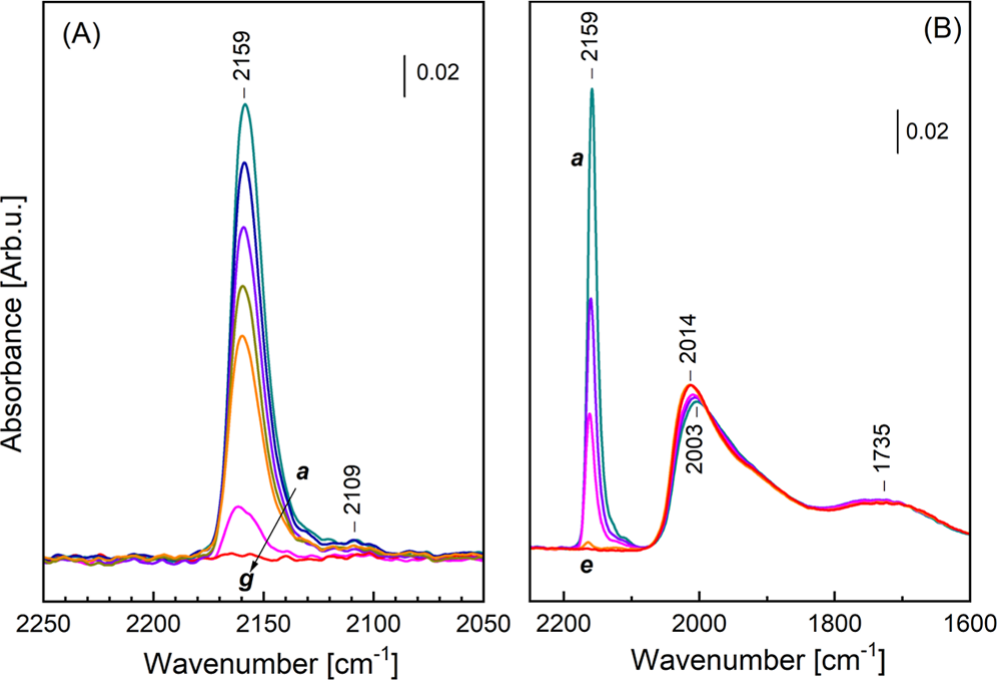
FTIR spectra of CO adsorbed at −173 °C on the Ru/CeO_2_ catalyst. (A)—Oxidized sample. (B)—Reduced sample. Initial CO equilibrium pressure of 2 mbar (a) and during decrease of the pressure in the conditions of dynamic vacuum (b–g).

No carbonyls of Ru^n+^ were observed, probably due to two reasons. First, the Ru^*n*+^ sites could be blocked by the OH groups. Second, carbonyls are not produced with cations with high oxidation state, as documented by numerous examples, e.g. V^5+^ and Mo^6+^.^14^ It should also be noted that, despite the relatively low concentration of loaded Ru, it significantly decreases the overall number of Ce^n+^ CO adsorption sites (Figures 6 vs [Fig fig8]). This indirectly testifies the assumption that oxidative treatment of Ru/CeO_2_ could lead to ruthenium ions migration from the Ru NPs to the surface of the CeO_2_ support and blocking the potential Ce^3+^ and Ce^4+^ adsorption sites of CO. This assumption agrees well with works of Liu et al.^7^ and Idriss et al.^55^ They reported that oxidative treatment of the Ru/CeO_2_ catalyst results in the redispersion of Ru on the ceria surface without any noticeable Ru dissolution into ceria. Taking into account the high reactivity of {110} and {100} faces of CeO_2_,^6,8^ they could be more favorable anchors than the low active {111} face. Nevertheless, the proposed mechanism of CO adsorption site blocking requires further experimental and theoretical verification.

On the reduced sample, the same band is observed but with slightly increased intensity at saturation (Figure 8B), indicating that reduction has liberated some Ce sites on the {111} facets—an effect often observed for reduced supported catalysts.^50^ In addition, a strong band at 2007 cm^–1^ and a weaker one at 1740 cm^–1^ appear, corresponding to CO adsorbed on metallic Ru in linear and bridged configurations, respectively.^56^ However, according to literature data, the band at 1740 cm^–1^ could also be due to μ-bonded carbonyls, in which both the carbon and oxygen atoms of the adsorbed CO are bonded to different Ru sites.^57^

Upon evacuation, the 2159 cm^–1^ band decreases in intensity and ultimately disappears, whereas the 2007 cm^–1^ band gains some intensity and shifts to 2013 cm^–1^. This shift arises from increased dipole–dipole coupling among the adsorbed CO molecules and, together with the intensity rise, suggests a slight increase in CO population on the metal. The effect appears to be steric in nature, arising from competition between CO adsorbed on Ru and on nearby Ce^4+^ sites.

These observations confirm that both metallic Ru and bare {111} CeO_2_ facets are exposed on the sample surface after reduction. It agrees well with the NAP-XPS data (see Figure 4).

Because CO adsorption on supported Ru at room temperature may lead to morphological or oxidative changes (“corrosive CO adsorption”)^19,20,48^ we tested the stability of the adsorbed species upon heating in the presence of CO (see Figure S5 in Supporting Information).

Increase of temperature leads to a fast disappearance of the band due to cerium carbonyls because of their low stability. Another effect is that the Ru^0^–CO band develops while shifting to 2029 cm^–1^. At room temperature, the band appears with a strongly enhanced intensity. This finding indicates CO-induced increase of dispersion of the Ru nanoparticles.^50^ Importantly, no oxidized Ru species (expected above 2100 cm^–1^) were detected. Thus, it appears that the presence of reduced cerium sites prevents the well-known CO-induced oxidation of metallic Ru particles, highlighting the role of the ceria support in modulating the redox chemistry of ruthenium.

Finally, we examined the possibility of the liberation of additional Ce^*n*+^ sites (particularly at the {110} and {111} facets) by increasing the reduction temperature. However, no additional Ce^*n*+^ sites were liberated even after reduction at 500 °C (Figure S6).

### Adsorption of ^15^N_2_ on Oxidized and Reduced Ru/CeO_2_

No ^15^N_2_ adsorption bands were observed on Ru/CeO_2_, irrespective of the sample pretreatment (see Figure S7 in Supporting Information). This is consistent with the CO results showing that the {100} and {110} facets—where N_2_ adsorption occurs—are practically blocked by the supported Ru nanoparticles.

Interestingly, no ^15^N_2_ adsorbed on metallic ruthenium was detected. In a recent study Akrour et al.^58^ detected bands around 2210 cm^–1^ after adsorption of ^14^N_2_ on 2 nm Ru nanoparticles supported on hydroxyapatite. These bands correspond to ^15^N_2_ bands around 2135 cm^–1^. Therefore, very low intensity bands in the region could have been masked by the Ce^3+^ electronic transition band. However, the absence of a clear signal, coupled with the fact that Ru^0^ sites were detected with CO, indicates that, as with ceria support, ^15^N_2_ does not probe all accessible Ru^0^ sites but only the strongest ones. This is consistent with the higher ruthenium dispersion of the samples studied by Akrour et al.^58^

## Conclusions

The Ru/CeO_2_ sample was synthesized using a wet-chemistry technique. XRD and STEM analyses indicate that it consists of Ru nanoparticles approximately 10 nm in size, supported on CeO_2_ nanooctahedra that are predominantly terminated by {111} faces. EDS mapping shows that almost all ruthenium is concentrated in the Ru nanoparticles. Using the NAP-XPS technique, we showed that the oxidative treatment (5 mbar O_2_ at 300 °C) of the Ru/CeO_2_ sample results in almost complete Ce^3+^ oxidation to Ce^4+^ and oxidation of most of the Ru to Ru^4+^ and Ru^*n*+^(*n* ≥ 4+) forms. The reductive treatment (5 mbar H_2_ at 350 °C) results in a noticeable reduction of ceria support and complete reduction of Ru nanoparticles to the Ru^0^ state. Next, the oxidized and reduced CeO_2_ and Ru/CeO_2_ samples were studied by IR technique. Ru/CeO_2_ treated in air at 300 °C exhibits a background spectrum that includes a weak Ce^3+^ feature comparable to that of bare ceria and a broad halo of H-bonded OH groups associated with Ru. No bands assignable to Ru=O species are observed, indicating the absence of crystalline RuO_2_ species, despite a high degree of Ru oxidation observed by the NAP-XPS technique. CO and ^15^N_2_ probing of the Ru/CeO_2_ sample allows us to draw the following conclusions.

(i) Ru loading significantly decreases the overall number of CO adsorption sites and completely blocks ^15^N_2_ adsorption on CeO_2_.
(ii) On the oxidized Ru/CeO_2_, all Ce^4+^ sites on {110} and {100} facets are fully blocked by ruthenium, and the spectrum is dominated by a single feature at 2159 cm^–1^ from CO coordinated to Ce sites on the {111} surface.
(iii) On the reduced Ru/CeO_2_ sample, CO adsorption reveals both linear and bridged CO on metallic Ru (2007 and 1740 cm^–1^), as well as slightly increased accessibility of Ce sites on the {111} facets. Upon evacuation, the Ru–CO band slightly rises and shifts to 2013 cm^–1^, indicating enhanced dynamic interactions due to a minor increase in CO population on Ru.
(iv) Heating the sample in CO induces restructuring of Ru nanoparticles, but no oxidized Ru^*n*+^–CO species form, highlighting the stabilizing influence of the ceria support on Ru^0^.
(v) No ^15^N_2_ adsorption bands are observed for the Ru/CeO_2_, regardless of pretreatment.

## Supplementary Material



## Notes

Structural parameters of CeO_2_ and Ru/CeO_2_ samples; experimental and calculated values of H_2_ consumption by CeO_2_ and Ru/CeO_2_ samples; Ce 3d and Ru 3d region of the NAP-XPS spectra of Ru/CeO_2_ sample; effect of evacuation temperature on the spectrum of oxidized Ru/CeO_2_; FTIR spectra of activated CeO_2_ nanoparticles with cubic morphology before and after calcination; shift of the OH modes; FTIR spectra of CO adsorbed on the Ru/CeO_2_ catalyst upon heating up from- 173 °C to room temperature; FTIR spectra of CO adsorbed on the Ru/CeO_2_ catalyst after reduction; FTIR spectra of ^15^N_2_ adsorbed at −173 °C on oxidized and reduced Ru/CeO_2_ sample (PDF)

## References

[1] Hu Z., Wang Z., Guo Y., Wang L., Guo Y., Zhang J., Zhan W. (2018). Total Oxidation of Propane over a
Ru/CeO_2_ Catalyst at Low Temperature. Environ. Sci. Technol..

[2] Bezkrovnyi O., Divins N. J., Serrano I., Garcia X., Kraszkiewicz P., Ptak M., Pawlyta M., Kępiński L., Llorca J. (2025). Anticoking Effect of Eu^3+^ Doping of the
Ru/Ceria Catalyst in the MSR Reaction for Hydrogen Generation. J. Phys. Chem. C.

[3] Qin X., Chen X., Chen M., Zhang J., He H., Zhang C. (2021). Highly Efficient
Ru/CeO_2_ Catalysts for Formaldehyde Oxidation
at Low Temperature and the Mechanistic Study. Catal. Sci. Technol..

[4] Mudiyanselage K., Al-Shankiti I., Foulis A., Llorca J., Idriss H. (2016). Reactions
of Ethanol over CeO_2_ and Ru/CeO_2_ Catalysts. Appl. Catal., B.

[5] López-Rodríguez S., Davó-Quiñonero A., Bailón-García E., Lozano-Castelló D., Bueno-López A. (2021). Effect of
Ru Loading on Ru/CeO_2_ Catalysts for CO_2_ Methanation. Mol. Catal..

[6] Trovarelli A., Llorca J. (2017). Ceria Catalysts at
Nanoscale: How Do Crystal Shapes
Shape Catalysis?. ACS Catal..

[7] Liu P., Zheng C., Liu W., Wu X., Liu S. (2024). Oxidative
Redispersion-Derived Single-Site Ru/CeO_2_ Catalysts with
Mobile Ru Complexes Trapped by Surface Hydroxyls Instead of Oxygen
Vacancies. ACS Catal..

[8] Aitbekova A., Wu L., Wrasman C. J., Boubnov A., Hoffman A. S., Goodman E. D., Bare S. R., Cargnello M. (2018). Low-Temperature
Restructuring of
CeO_2_-Supported Ru Nanoparticles Determines Selectivity
in CO_2_ Catalytic Reduction. J. Am.
Chem. Soc..

[9] Bezkrovnyi O., Vorokhta M., Pawlyta M., Ptak M., Piliai L., Xie X., Dinhová T. N., Khalakhan I., Matolínová I., Kepinski L. (2022). In Situ Observation
of Highly Oxidized Ru Species in Ru/CeO_2_ Catalyst under
Propane Oxidation. J. Mater. Chem. A.

[10] Dinhová T. N., Bezkrovnyi O., Piliai L., Khalakhan I., Chakraborty S., Ptak M., Kraszkiewicz P., Vaidulych M., Mazur M., Vajda S. ˇ., Kepinski L., Vorochta M., Matolínová I. (2025). Unraveling
the Effects of Reducing and Oxidizing Pre-treatments and Humidity
on the Surface Chemistry of the Ru/CeO_2_ Catalyst during
Propane Oxidation. J. Phys. Chem. C.

[11] Xu K., Hu X. C., Ma C., Wang P., Wang W. W., Jia C. J. (2023). Spectroscopic Investigation of the Structural Transformation
of Ru in the Ru/CeO_2_ Catalyst. Catal.
Sci. Technol..

[12] Khivantsev K., Jaegers N. R., Aleksandrov H. A., Song I., Pereira-Hernandez X. I., Engelhard M. H., Tian J., Chen L., Motta Meira D., Kovarik L., Vayssilov G. N., Wang Y., Szanyi J. (2023). Single Ru­(II)
Ions on Ceria as a Highly Active Catalyst for Abatement of NO. J. Am. Chem. Soc..

[13] Bezkrovnyi O., Kraszkiewicz P., Vorochta M. (2024). In Situ Study of the Effect of the
Exposed Surface of Ceria (100 vs 111) on the Highly Oxidized Species
Formation on Ru/Ceria Catalysts. Acta Phys.
Polym., A.

[14] Hadjiivanov K., Vayssilov G. (2002). Characterization
of Oxide Surfaces and Zeolites by
Carbon Monoxide as an IR Probe Molecule. Adv.
Catal..

[15] Bezkrovnyi O., Bruix A., Blaumeiser D., Piliai L., Schötz S., Bauer T., Khalakhan I., Skála T., Matvija P., Kraszkiewicz P., Pawlyta M., Vorokhta M., Matolínová I., Libuda J., Neyman K. M., Kȩpiński L. (2022). Metal-Support
Interaction and Charge
Distribution in Ceria-Supported Au Particles Exposed to CO. Chem. Mater..

[16] Wang Y., Liu Z., Confer M. P., Li J., Wang R. (2021). In-Situ DRIFTS Study
of Chemically Etched CeO_2_ Nanorods Supported Transition
Metal Oxide Catalysts. Mol. Catal..

[17] Li C., Sakata Y., Arai T., Domen K., Maruya K.-I., Onishi T. (1989). Adsorption of Carbon Monoxide and Carbon Dioxide on
Cerium Oxide Studied by Fourier-Transform Infrared Spectroscopy Part
2.—Formation of Formate Species on Partially Reduced CeO2 at
Room Temperature. J. Chem. Soc. Faraday Trans.
1 Phys. Chem. Condens. Phases.

[18] Cao T., You R., Li Z., Zhang X., Li D., Chen S., Zhang Z., Huang W. (2020). Morphology-Dependent CeO_2_ Catalysis in Acetylene Semihydrogenation. Appl. Surf. Sci..

[19] Yokomizo G. H., Louis C., Bell A. T. (1989). An Infrared
Study of CO Adsorption
on Reduced and Oxidized Ru/SiO_2_. J. Catal..

[20] Solymosi F., Raskó J. (1989). An Infrared
Study of the Influence of CO Adsorption
on the Topology of Supported Ruthenium. J. Catal..

[21] Chakarova K. K., Mihaylov M. Y., Karapenchev B. S., Drenchev N. L., Ivanova E. Z., Vayssilov G. N., Aleksandrov H. A., Hadjiivanov K. I. (2025). FTIR Detection
of Ce^3+^ Sites on Shape-Controlled Ceria Nanoparticles Using
Adsorbed ^15^N_2_ as a Probe Molecule. Molecules.

[22] Chakarova K. K., Mihaylov M. Y., Karapenchev B. S., Koleva I. Z., Vayssilov G. N., Aleksandrov H. A., Hadjiivanov K. I. (2024). N_2_ as an Efficient IR
Probe Molecule for the Investigation of Ceria-Containing Materials. Molecules.

[23] Bezkrovnyi O., Kraszkiewicz P., Miśta W., Kepinski L. (2020). The Sintering of Au
Nanoparticles on Flat {100}, {111} and Zigzagged {111}-Nanofacetted
Structures of Ceria and Its Influence on Catalytic Activity in CO
Oxidation and CO PROX. Catal. Lett..

[24] Bezkrovnyi O., Kraszkiewicz P., Ptak M., Kepinski L. (2018). Thermally Induced Reconstruction
of Ceria Nanocubes into Zigzag {111}-Nanofacetted Structures and Its
Influence on Catalytic Activity in CO Oxidation. Catal. Commun..

[25] Rodriguez-Carvajal J. (1993). Recent Advances
in Magnetic Structure Determination Neutron Powder Diffraction. Phys. B Cond. Matter.

[26] Bezkrovnyi O. (2025). Comprehensive
H_2_-TPR Study of the Lanthanide Oxides Reducibility. J. Clust. Sci..

[27] Bezkrovnyi O., Kraszkiewicz P., Vorochta M. (2025). The Effect of H_2_ Pressure
on the Reduction of CeO_2_ Nanocrystals. Low Temp. Phys..

[28] Chakarova K., Drenchev N., Mihaylov M., Hadjiivanov K. (2024). Interaction
of O_2_ with Reduced Ceria Nanoparticles at 100–400
K: Fast Oxidation of Ce^3+^ Ions and Dissolved H_2_. Catalysts.

[29] Rao G. R. (1999). Influence
of Metal Particles on the Reduction Properties of Ceria-Based Materials
Studied by TPR. Bull. Mater. Sci..

[30] Boaro M., Vicario M., De Leitenburg C., Dolcetti G., Trovarelli A. (2003). The Use of
Temperature-Programmed and Dynamic/Transient Methods in Catalysis:
Characterization of Ceria-Based, Model Three-Way Catalysts. Catal. Today.

[31] Lee J., Ryou Y., Chan X., Kim T. J., Kim D. H. (2016). How Pt
Interacts with CeO_2_ under the Reducing and Oxidizing Environments
at Elevated Temperature: The Origin of Improved Thermal Stability
of Pt/CeO_2_ Compared to CeO_2_. J. Phys. Chem. C.

[32] Wang H., Luo S., Zhang M., Liu W., Wu X., Liu S. (2018). Roles of Oxygen
Vacancy and Ox^–^ in Oxidation Reactions over CeO_2_ and Ag/CeO_2_ Nanorod Model Catalysts. J. Catal..

[33] Yao H. C., Yao Y. F. Y. (1984). Ceria in Automotive
Exhaust Catalysts I. Oxygen Storage. J. Catal..

[34] Bezkrovnyi O., Małecka M. A., Lisiecki R., Ostroushko V., Thomas A. G., Gorantla S., Kepinski L. (2018). The Effect of Eu Doping
on the Growth, Structure and Red-Ox Activity of Ceria Nanocubes. CrystEngComm.

[35] Bezkrovnyi O., Szymczak M., Marciniak L., Seminko V., Kraszkiewicz P., Małecka M., Pawlyta M., Vorochta M., Matolínová I. (2025). Advances and
Limitations of the Eu^3+^ Luminescent Probe for Monitoring
Ce^4+^/Ce^3+^ Transitions in Ceria. J. Phys. Chem. C.

[36] Skala T., Sutara F., Prince K. C., Matolín V. (2009). Journal of
Electron Spectroscopy and Cerium Oxide Stoichiometry Alteration via
Sn Deposition: Influence of Temperature. J.
Electron Spectrosc. Relat. Phenom..

[37] Bezkrovnyi O. S., Blaumeiser D., Vorokhta M., Kraszkiewicz P., Pawlyta M., Bauer T., Libuda J., Kepinski L. (2020). NAP-XPS and
In Situ DRIFTS of the Interaction of CO with Au Nanoparticles Supported
by Ce_1–x_Eu_x_O_2_ Nanocubes. J. Phys. Chem. C.

[38] Bezkrovnyi O. S., Mykhailo V., Małgorzata A. M., Miśta W., Kepinski L. (2020). NAP-XPS Study of Eu^3+^→Eu^2+^ and Ce^4+^→Ce^3+^ Reduction in Au/Ce_0.80_Eu_0.20_O_2_ Catalyst. Catal. Commun..

[39] Chakarova K. K., Zdravkova V. R., Karapenchev B. S., Nihtianova D. D., Ivanova E. Z., Aleksandrov H. A., Koleva I. Z., Panayotov D. A., Mihaylov M. Y., Vayssilov G. N., Hadjiivanov K. I. (2024). Evolution
of Ce^4+^ Lewis Acidity during Dehydroxylation of Ceria Nanoparticles
with Different Morphology: An Integrated FTIR, DFT and HRTEM Study. J. Catal..

[40] Chakarova K. K., Karapenchev B. S., Drenchev N. L., Ivanova E. Z., Aleksandrov H. A., Panayotov D. A., Mihaylov M. Y., Vayssilov G. N., Hadjiivanov K. I. (2025). FTIR Study of Low-Temperature CO Adsorption on Reduced
Ceria Nanoparticles with Different Morphology: A Comparison with Oxidized
Samples. J. Catal..

[41] Bernal S., Calvino J. J., Cifredo G. A., Gatica J. M., Omil J. A. P., Pintado J. M. (1993). Hydrogen Chemisorption on Ceria: Influence of the Oxide
Surface Area and Degree of Reduction. J. Chem.
Soc., Faraday Trans..

[42] Badri A., Binet C., Lavalley J.-C. (1996). An FTIR
Study of Surface Ceria Hydroxy
Groups during a Redox Process with H_2_. J. Chem. Soc., Faraday Trans..

[43] Binet C., Badri A., Lavalley J.-C. (1994). A Spectroscopic Characterization
of the Reduction of Ceria from Electronic Transitions of Intrinsic
Point Defects. J. Phys. Chem..

[44] Binet C., Daturi M., Lavalley J.-C. (1999). IR Study of Polycrystalline
Ceria
Properties in Oxidised and Reduced States. Catal.
Today.

[45] Bozon-Verduraz F., Bensalem A. (1994). IR Studies of Cerium
Dioxide: Influence of Impurities
and Defects. J. Chem. Soc., Faraday Trans..

[46] Afrin S., Bollini P. (2023). On the Utility of Ce^3+^ Spin–Orbit
Transitions in the Interpretation of Rate Data in Ceria Catalysis:
Theory, Validation, and Application. J. Phys.
Chem. C.

[47] Mandel G., Bauman R. P., Banks E. (1960). Electronic
Transitions of Rare Earth
Ions in the Infrared Region. J. Chem. Phys..

[48] Tong X., Luo T., Meng X., Wu H., Li J., Liu X., Ji X., Wang J., Chen C., Zhan Z. (2015). Shape-Dependent Activity
of Ceria for Hydrogen Electro-Oxidation in Reduced-Temperature Solid
Oxide Fuel Cells. Small.

[49] Butova V., Drenchev N. L., Hadjiivanov K. I. (2026). In Situ
FTIR Investigation of Metal
Node Chemistry in Zr-Ce-and Zr-Ce Bimetallic UiO-66 Frameworks. Precis. Chem..

[50] Hadjiivanov K., Lavalley J.-C., Lamotte J., Maugé F., Saint-Just J., Che M. (1998). FTIR Study of CO Interaction with
Ru/TiO_2_ Catalysts. J. Catal..

[51] Hadjiivanov K., Saint-Just J., Che M., Tatibouet J.-M., Lamotte J., Lavalley J.-C. (1994). Preparation and Characterization
of Multiple Ion-Exchanged Pt/TiO_2_ Catalysts. J. Chem. Soc. Faraday Trans..

[52] Yang C., Wöll C. (2024). Infrared Reflection-Absorption Spectroscopy (IRRAS)
Applied to Oxides: Ceria as a Case Study. Surf.
Sci..

[53] Rojas-Buzo S., Concepción P., Olloqui-Sariego J. L., Moliner M., Corma A. (2021). Metalloenzyme-Inspired
Ce-MOF Catalyst for Oxidative Halogenation Reactions. ACS Appl. Mater. Interfaces.

[54] Farra R., Wrabetz S., Schuster M. E., Stotz E., Hamilton N. G., Amrute A. P., Pérez-Ramírez J., López N., Teschner D. (2013). Understanding CeO_2_ as
a Deacon Catalyst by Probe Molecule Adsorption and in Situ Infrared
Characterisations. Phys. Chem. Chem. Phys..

[55] Idriss H., Llorca J. (2019). Low Temperature Infrared
Study of Carbon Monoxide Adsorption
on Rh/CeO_2_. Catalysts.

[56] Conesa J. C. (1995). Computer
Modeling of Surfaces and Defects on Cerium Dioxide. Surf. Sci..

[57] Xu S., Chansai S., Xu S., Stere C. E., Jiao Y., Yang S., Hardacre C., Fan X. (2020). CO Poisoning of Ru
Catalysts in CO_2_ Hydrogenation under Thermal and Plasma
Conditions: A Combined Kinetic and Diffuse Reflectance Infrared Fourier
Transform Spectroscopy-Mass Spectrometry Study. ACS Catal..

[58] Akrour S., Vimont A., Costentin G., Thomas C. (2025). N_2_ as a
Quantitative FTIR Probe Molecule for Reliable Assessment of the Dispersion
of Ru Supported on Hydroxyapatite. J. Phys.
Chem. C.

