# Supplementary material for: Surface Chemistry of Ru/CeO2 Catalyst as Revealed by CO and 15N2 IR Probe Molecules
Source: Langmuir. 2026 Apr 23;42(17):12238–47. doi: 10.1021/acs.langmuir.6c00814 (PMC13309001; doi:10.1021/acs.langmuir.6c00814)
Supplement: Supplementary file 1 [file la6c00814_si_001.pdf]

Supporting Information (SI)

Surface chemistry of Ru/CeO_2 catalyst as revealed by CO and $^{15}N_2$ IR probe molecules

Oleksii Bezkrovnyi,[1*] Nikola Drenchev,[2,3] Piotr Kraszkiewicz,[1] Michael Vorochta,[4] Iva Matolínová,[4] Tcvetomir Venkov,[2] Mirosława Pawłyta,[5,6] Leszek Kepinski[1] and Konstantin Hadjiivanov[2,3*]

1. W. Trzebiatowski Institute of Low Temperature and Structure Research, Polish Academy of Sciences, 50-422 Wroclaw, Poland
2. Institute of General and Inorganic Chemistry, Bulgarian Academy of Sciences, 1113 Sofia, Bulgaria
3. Centre of Mechatronics and Clean Technologies, Sitnyakovo Campus, 1113 Sofia, Bulgaria.
4. Department of Surface and Plasma Science, Faculty of Mathematics and Physics, Charles University, V Holešovičkách 2, Prague 8 180 00, Czech Republic
5. Materials Research Laboratory, Silesian University of Technology, Gliwice 44-100, Poland
6. Institute of Nuclear Physics, Polish Academy of Sciences, str.. Radzikowskiego 152 31-342 Kraków, Poland

*Corresponding authors: o.bezkrovnyi@intibs.pl; kih@svr.igic.bas.bg

Table S1 –Structural parameters of CeO_2 and Ru/CeO_2 samples calculated using FullProf.

**Sample**	**Average cryst. size, [nm]**	**Average strain *10^{-4}**	**Unit cell parameters**						
			**a, [nm]**	**b, [nm]**	**c, [nm]**	**α, [°]**	**β, [°]**	**γ, [°]**	**Space group**
**CeO_2**	37.04 +/-0.02	4.50 +/- 0.01	0.54134 +/- 0.00005	0.54134	0.54134	90	90	90	*Fm-3m*
**As-received Ru/CeO_2**	CeO_2 phase								
	40.82 +/-0.02	4.00 +/- 0.01	0.54146 +/- 0.00004	0.54146	0.54146	90	90	90	*Fm-3m* 98.0% +/-0.1%
	Ru (hex) phase								
	14.02 +/-0.01	n.d.	0.2707 +/-0.0006	0.2707	0.4285 +/- 0.0009	90	90	120	*$P6_3/mmc$* 3.36% +/-0.2%
**Oxidized Ru/CeO_2**	CeO_2 phase								
	38.74 +/-0.02	4.33 +/- 0.01	0.54125 +/- 0.00006	0.54125	0.54125	90	90	90	*Fm-3m*
**Reduced Ru/CeO_2**	CeO_2 phase								
	51.55 +/-0.02	6.72 +/- 0.03	0.54155 +/- 0.00003	0.54155	0.54155	90	90	90	*Fm-3m* 97.16% +/-0.37%
	Ru (hex) phase								
	15.14 +/-0.01	n.d.	0.2708 +/-0.0006	0.2708	0.4279 +/- 0.0009	90	90	120	*$P6_3/mmc$* 2.84% +/-0.02%

Note: The standard deviations appearing in the global average apparent size and strain are calculated using the different reciprocal lattice directions. It is a measure of the degree of anisotropy, not of the estimated error.

Table S2. Experimental and calculated values of H_2 consumption by CeO_2 and Ru/CeO_2 samples.

Sample	H_2 consumption [μmol/50mg] – measured			H_2 consumption [μmol/50mg] – theoretical [a]		
	0 – 300°C	0 – 450 °C	0 – 1000 °C	RuO_2	Support	Catalyst (total)
CeO_2	0.75	4.68	58.80	—	145.2	145.2
Ru/CeO_2	20.66	21.89	77.19	26.7	140.1	166.8

a Assuming total reduction of RuO_2 and total reduction of Ce^{4+} to Ce^{3+}, for a fully oxidized catalyst.

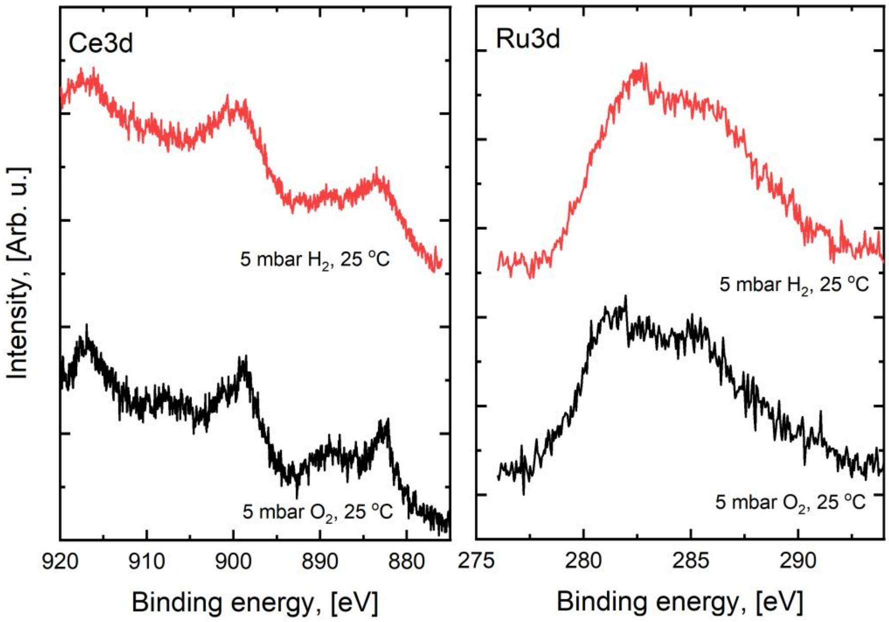

Figure S1. Ce3d and Ru 3d region of the NAP-XPS spectra of Ru/CeO_2 sample.

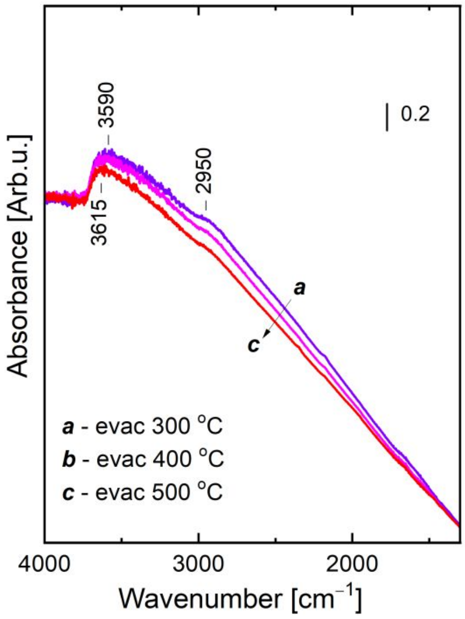

Figure S2. Effect of evacuation temperature on the spectrum of oxidized Ru/CeO_2.

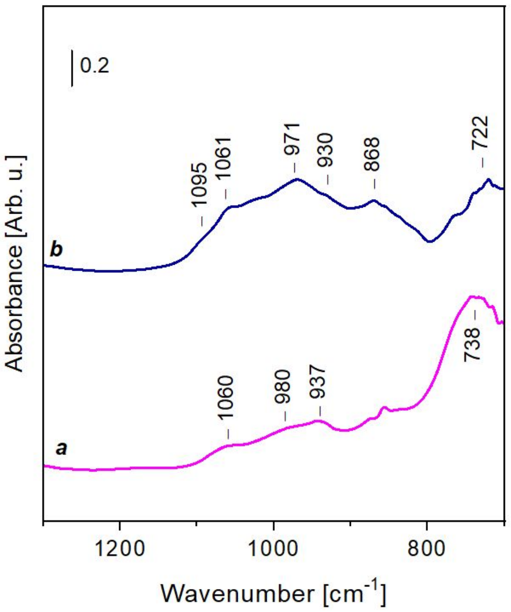

Figure S3. FTIR spectra of activated CeO_2 nanoparticles with cubic morphology (for details see ref. 39 in the main text) (a) and after calcination of the sample at 650 ^oC (b).

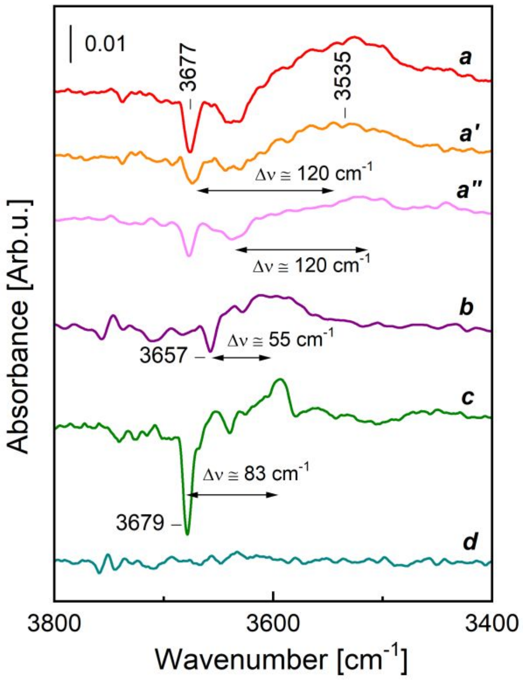

Figure S4. Shift of the OH modes. a - oxidized ceria with CO, a' step 1, a'' step 2; b - reduced ceria with CO; c -oxidized ceria with N_2; reduced ceria with N_2.

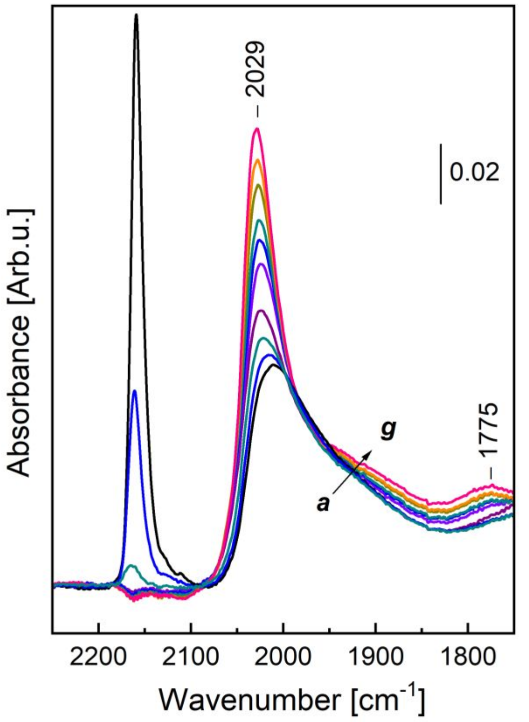

Figure S5. FTIR spectra of CO adsorbed on the Ru/CeO_2 catalyst upon heating up from- 173 °C to room temperature at 2 mbar of CO

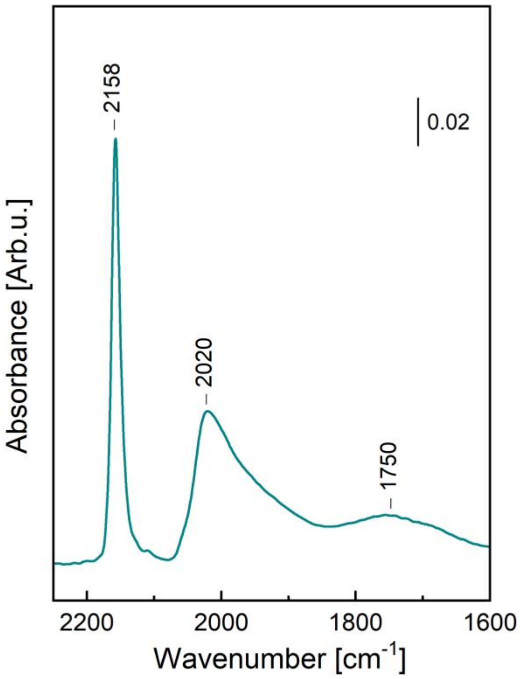

Figure S6. FTIR spectra of CO adsorbed on the Ru/CeO_2 catalyst after reduction in 50 mbar of H_2 at 500 °C

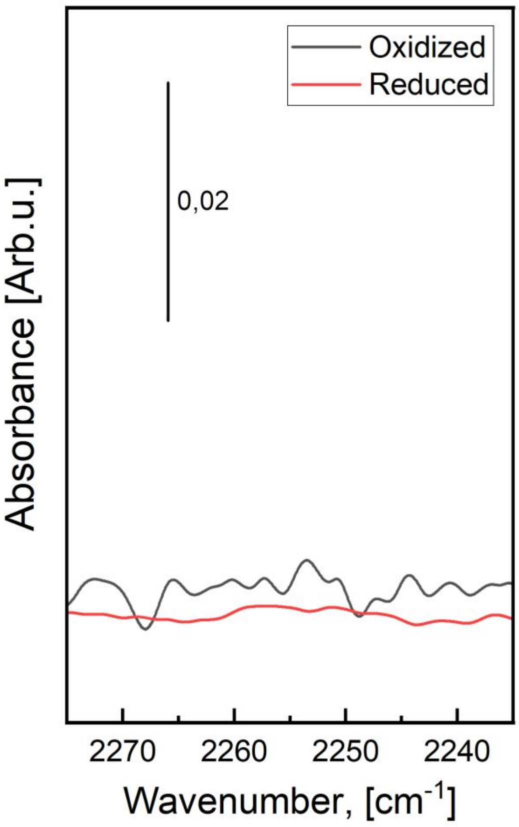

Figure S7. FTIR spectra of $^{15}N_2$ adsorbed at -173 °C on oxidized and reduced Ru/CeO_2 sample. Equilibrium pressure of $^{15}N_2$ of 5.0 mbar.
